# Tear matrix metalloproteinase 9 immunoassay positivity reflects the severity of conjunctivochalasis

**DOI:** 10.1038/s41598-025-95195-4

**Published:** 2025-10-13

**Authors:** Jiyeon Lee, Seung Pil Bang, Jaekyoung Lee, Kyu Young Shim, Jong Hwa Jun

**Affiliations:** 1 Keimyung University School of Medicine, Daegu, South Korea; 2 Department of Ophthalmology, Keimyung University School of Medicine, 1035 Dalgubeol-daero, Dalseo-gu, Daegu, 42601 Republic of Korea

**Keywords:** Conjunctivochalasis, InflammaDry, Matrix metalloproteinase-9, Point-of-care, Tears, Biomarkers, Conjunctival diseases, Lacrimal apparatus diseases

## Abstract

An objective indicator for diagnosing and evaluating the therapeutic efficacy of conjunctivochalasis (CCh) treatments is not available. Therefore, we investigated the correlation between CCh severity and the matrix metalloproteinase-9 (MMP)-9 point-of-care (POC) semi-quantitative test to determine its suitability for diagnosing and evaluating CCh. We conducted a prospective study comprising healthy participants and patients with evaporative dry eyes and CCh. The participants underwent CCh severity, tear break-up time, Schirmer test, ocular staining score, tear meniscus height, and tear MMP-9 evaluations. The MMP-9 test results were classified into negative and weakly, moderately, and strongly positive. We included 108 participants; 54 participants had CCh and 54 did not. The MMP-9 positivity rate was significantly higher for patients with ≥ CCh grade 2 than those without CCh (*p* < 0.001). Of the CCh-negative participants, 75.9% were MMP-9-negative, and 24.1% were moderately/strongly positive. Conversely, 37.0% of CCh-positive patients were MMP-9-negative, and 63.0% were moderately/strongly positive. Furthermore, MMP-9 positivity significantly differed among the CCh severity groups (*p* < 0.001). Finally, there was a positive correlation between CCh severity and MMP-9 positivity (*p* < 0.001, *R* = 0.420). The tear MMP-9 positivity correlated with CCh severity. Therefore, the MMP-9 POC test may help diagnose CCh and evaluate its severity after treatment.

## Introduction

Conjunctivochalasis (CCh) is a common condition characterized by loose, redundant, non-edematous conjunctival folds primarily in the inferior bulbar area of the eyes. Chalatic conjunctiva primarily affects older people with clinical manifestations such as repetitive irritation, epiphora, and blurred vision due to secondary early tear break-up that induces chronic ocular discomfort^1–3^. The range of symptoms varies from mild discomfort in the early stage, to tear outflow impediment in the moderate stage, and finally, exposed ocular surfaces with subsequent vision deterioration in the severe stage^1,4–6^.

A definitive diagnosis is made when redundant conjunctival folds are observed in the inferior bulbar conjunctiva during slit-lamp examination. In addition, vital staining during the slit-lamp examination, such as with fluorescein dye, helps visualize the redundant conjunctival folds and tear film disturbances^1^. Nevertheless, determining clinically significant CCh can be challenging owing to other vague symptoms, such as ocular dryness, chronic irritation, discomfort, blurred vision, and ocular fatigue. These symptoms are often accompanied by other ocular surface diseases, such as dry eye disease (DED), making it more difficult to choose a CCh treatment. After a CCh diagnosis, medical therapy is generally attempted first to improve tear function and ocular surface inflammation^1,5^. The primary treatment options include tear substitutes, topical lubricants, and anti-inflammatory medications^5^.

Matrix metalloproteinase (MMP)-9 is a proteolytic enzyme produced by the corneal and conjunctival epithelium^7^. Previous studies have reported positive correlations between increased tear MMP-9 levels (both active and pro-forms) and DE disease severity^6,7^ and keratoconus^8^. Recent experimental studies also identified inflammatory components in CCh tears, which were positively correlated with an increased tear pro-MMP-9 level. Furthermore, the tear pro-MMP-9 level decreased after CCh surgery^9,10^. Therefore, monitoring the tear MMP-9 concentration may help evaluate the CCh treatment response or determine the necessity of a surgical intervention^11^.

The MMP-9 enzyme-linked immunosorbent assay and in-situ immunoassay (i.e. InflammaDry; Quidel, San Diego, CA) are popular methods for identifying elevated MMP-9 levels in tears. The former is a quantitative method that measures the MMP-9 concentration, but it is not reasonable for clinical practice. The latter is a qualitative method using immunoassays that can be performed in an office setting to detect MMP-9 concentrations over 40 ng/mL, measuring both the active and pro-forms of the MMP-9 on the ocular surface^12^.

Although the InflammaDry test was developed for qualitative evaluations, the method can also produce semi-quantitative results^7,11,13^. Therefore, we aimed to assess CCh severity using semi-quantitative evaluation with InflammaDry. Moreover, no objective or quantitative indicators exist for diagnosing and evaluating the therapeutic efficacy of surgery or medical treatments for CCh. Therefore, we also assessed the correlation between CCh severity and the semi-quantitative analysis results from the InflammaDry tear MMP-9 point-of-care (POC) test.

## Results

### Participants

We enrolled 108 participants (108 eyes); 54 eyes were CCh(–) (24 normal controls (NC) and 30 evaporative dry eyes (EDE)) and 54 were CCh(+). The mean age was 56.6 ± 15.3 years, and 63.9% were female (Male: Female = 39:69). The overall laterality was 60.2% (65 eyes) for the right eye and 39.8% (43 eyes) for the left eye.

### Ocular surface parameter comparisons

Table 1 presents the analysis of ocular surface parameters. Most parameters, including the ocular surface disease index (OSDI) score, Schirmer I test results, ocular staining scores (OSS), tear meniscus height (TMH), and MMP-9 positivity, showed no significant differences among the NC, EDE, and CCh groups, except for tear break-up time (TBUT) and MMP-9 positivity. TBUT differed significantly between the NC and EDE groups, while MMP-9 positivity did not. Additionally, ocular surface parameters were similar between the CCh(–) and CCh(+) groups, except for MMP-9 positivity.

**Table 1:** Ocular surface parameter comparisons between normal eyes and eyes with evaporative dry eye or conjunctivochalasis.

Parameters	Groups						*p*-value**	*p*-value***
	CCh (–)					CCh (+)		
	Normal		EDE	*p*-value*	Total			
OSDI scores	24.0 ± 21.3		32.5 ± 21.3	0.137	28.7 ± 21.6	36.0 ± 30.1	0.182	0.154
TBUT (seconds)	10.3 ± 2.6		4.1 ± 1.3	< 0.001	6.8 ± 3.7	6.2 ± 2.9	< 0.001	0.358
Schirmer I test (mm)	15.3 ± 6.3		16.7 ± 8.6	0.606	16.0 ± 7.6	14.7 ± 7.9	0.553	0.380
Fluorescein staining								
Corneal	0.38 ± 0.49		0.67 ± 0.92	0.146	0.53 ± 0.77	0.39 ± 0.66	0.186	0.284
Conjunctival (N)	0.21 ± 0.41		0.57 ± 0.90	0.061	0.41 ± 0.74	0.26 ± 0.48	0.052	0.221
Conjunctival (T)	0.21 ± 0.41		0.40 ± 0.67	0.124	0.31 ± 0.58	0.19 ± 0.39	0.146	0.175
TMH (µm)	319.1 ± 125.4		283.2 ± 99.7	0.186	299.1 ± 112.2	368.0 ± 249.1	0.150	0.068
MMP-9 positivity (%)	12.5		33.3	0.075	24.1	63.0	< 0.001	< 0.001

Values are presented as means ± standard deviations. Abbreviations: CCh: Conjunctivochalasis, EDE: Evaporative dry eye, N: Nasal, OSDI: Ocular surface disease index, T: Temporal, TBUT: Tear break up time, TMH: Tear meniscus height, MMP: matrix metalloproteinase.

*Normal and EDE group comparisons.

**Normal, EDE, and CCh (+) group comparisons.

***CCh (–) and CCh (+) group comparisons.

### CCh severity positively correlates with tear MMP-9 positivity

**Fig. 1:**
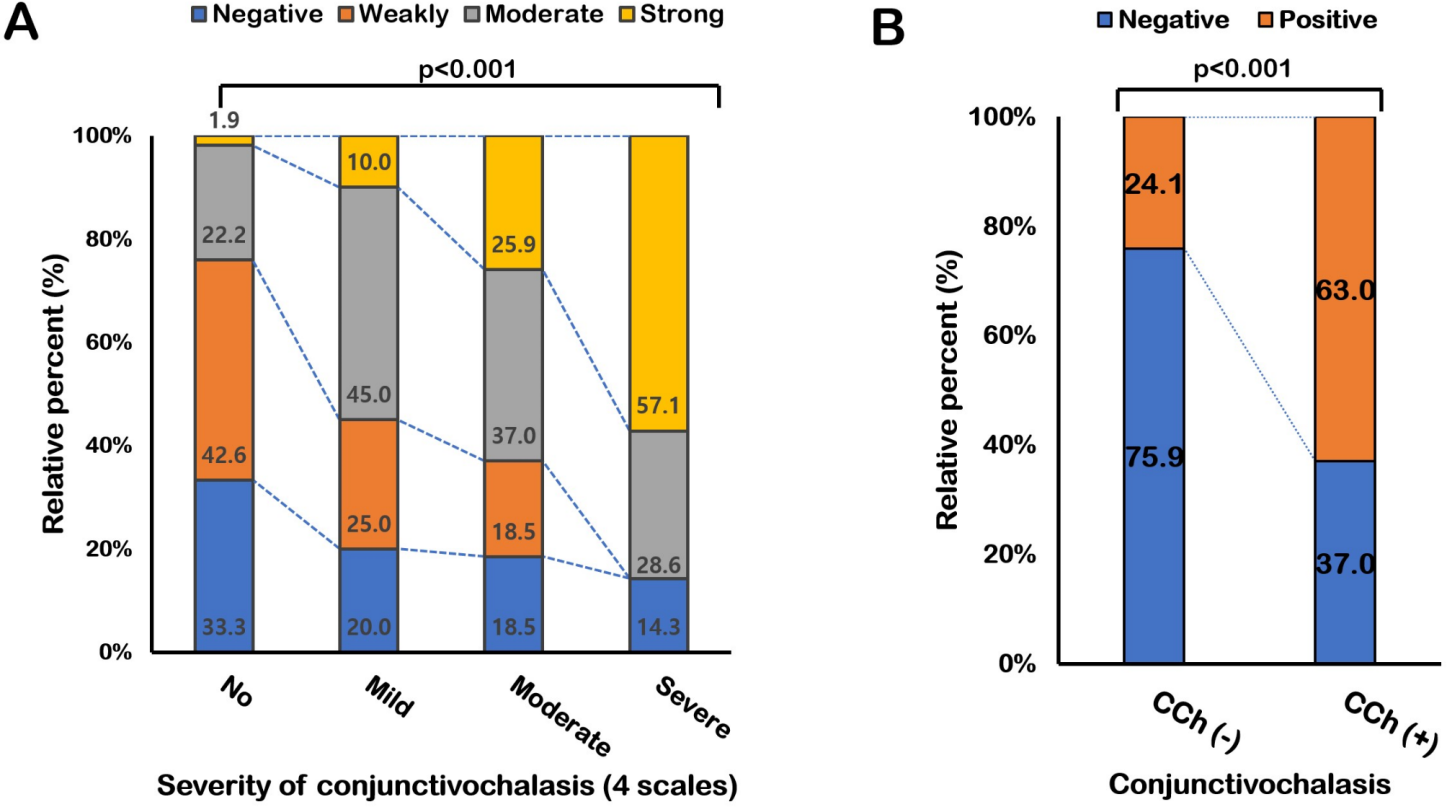
Conjunctivochalasis (CCh) severity positively correlates with tear matrix metalloproteinase (MMP)-9 positivity. (**A**) MMP-9 point-of-care (POC) test positivity from tears based on CCh severity. As CCh severity increased, the frequency of a moderately or strongly positive MMP-9 result increased (*p* < 0.001). (**B**) CCh (–) participants were more likely to have a negative MMP-9 test result than CCh (+) patients (77.8% vs. 37.0%; *p* < 0.001).

**Fig. 2:**
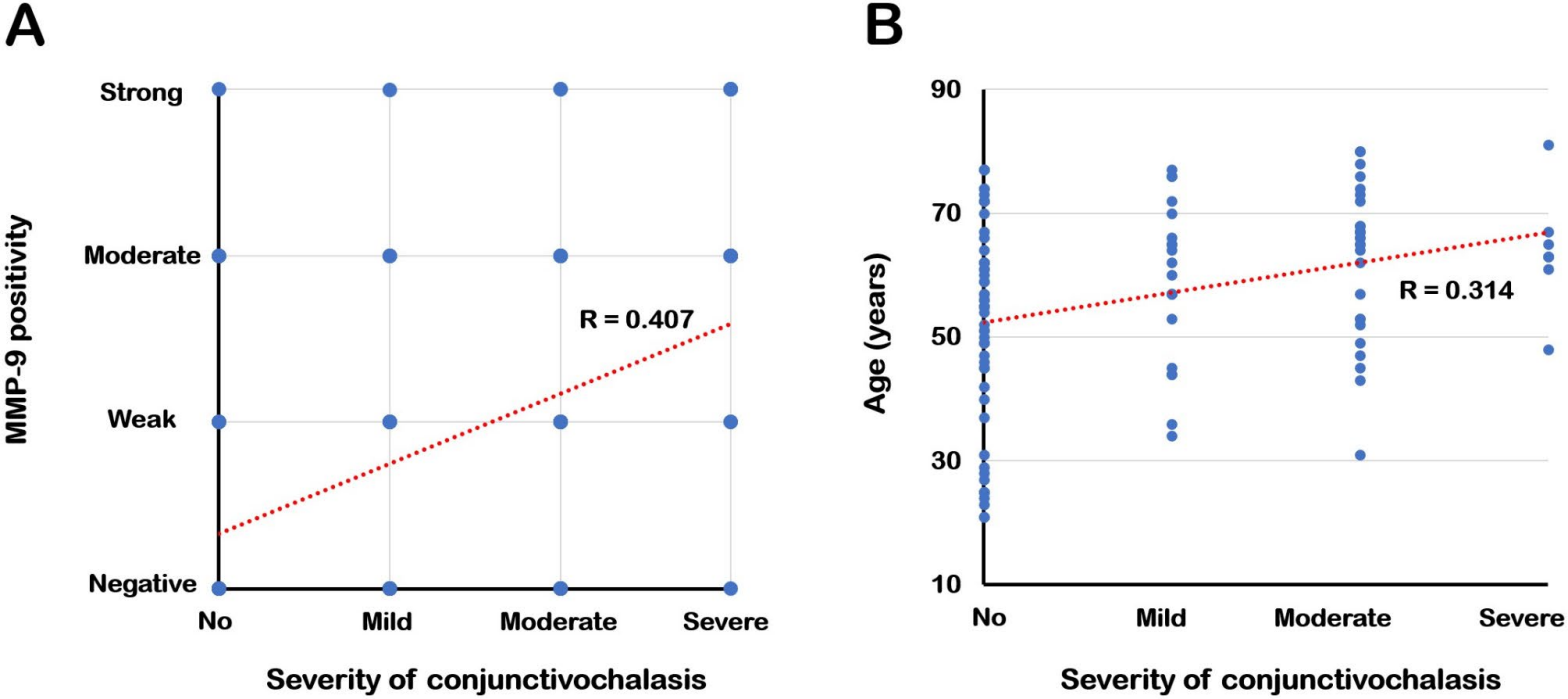
(**A**) Dot plot illustrating the correlation between conjunctivochalasis (CCh) severity and tear matrix metalloproteinase (MMP)-9 positivity, showing a positive linear relationship (*p* < 0.001, *R* = 0.421). (**B**) Dot plot illustrating the correlation between CCh severity and age, also demonstrating a positive linear relationship (*p* = 0.001, *R* = 0.314).

The MMP-9 positivity classification increased as the CCh severity increased (*p* < 0.001). More than 50% of CCh(+) participants were moderately or strongly MMP-9 positive. Of the CCh(–) participants, 24.1% were moderately or strongly positive, but only 1.9% were strongly positive (Fig. 1A). Moreover, participants in the CCh(+) group were MMP-9 positive significantly more frequently than those in the CCh(–) group (*p* < 0.001, Fig. 1B); 63.0% of participants in the CCh(+) group had an MMP-9 positive result, whereas only 24.1% of participants in the CCh(–) group had a positive result. The correlation analysis (including all participants) identified a correlation between MMP-9 positivity and CCh severity (*p* < 0.001, *R* = 0.421, Fig. 2A). Furthermore, CCh severity and age correlated (*p* = 0.001, *R* = 0.314, Fig. 2B), but other ocular surface parameters did not (*p* > 0.05).

## Diagnostic properties of the tear MMP-9 POC test for CCh

The overall sensitivity of the tear MMP-9 POC test (moderately/strongly positive) for diagnosing CCh was 63.0% (34/54), and the specificity was 77.8% (42/54). Furthermore, the accuracy, positive predictive value, and negative predictive value were 70.4% (76/108), 73.9% (34/46), and 67.7% (42/62), respectively.

**Fig. 3:**
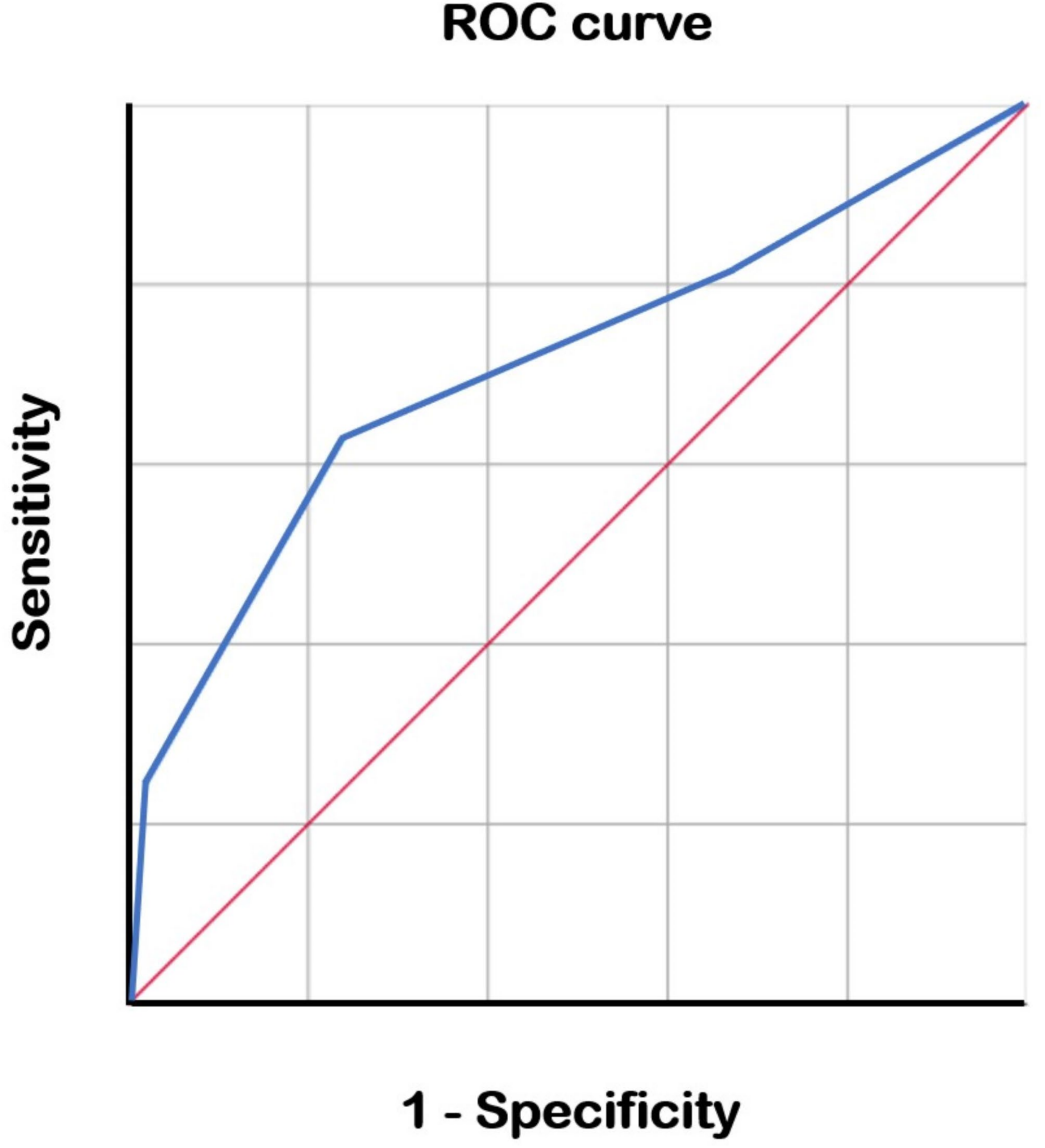
Receiver operating characteristic (ROC) curve demonstrating the diagnostic performance of the tear matrix metalloproteinase (MMP)-9 point-of-care test as a binary classifier for conjunctivochalasis (CCh). The area under the curve (AUC) is 0.709, indicating a 70.9% probability of accurately classifying CCh cases.

Moreover, we calculated the area under the receiver operating characteristic curve (Fig. 3) to determine an optimal cut-off value, finding that a strongly positive MMP-9 result suggested CCh with a sensitivity and specificity of 68.0% (34/50) and 75.9% (41/54), respectively. Furthermore, the positive predictive value was 72.3% (34/47), and the negative predictive value was 67.2% (41/61); the area under the curve was 0.709.

## Discussion

CCh is an aging process of the conjunctiva; thus, its prevalence increases rapidly with age^14–16^. Redundant and folded conjunctiva, which are characteristic of CCh, induce repeated subconjunctival hemorrhage, conjunctival injection, and symptoms of foreign body sensation, dryness, blurry vision, eye fatigue, and ocular burning. Nevertheless, not all patients require treatment. After a CCh diagnosis, medical treatment provides relief in most low-grade cases. However, for moderate or severe CCh cases, the response to medical treatment is usually low. Furthermore, it is particularly challenging to determine whether the patient’s discomfort is primarily caused by CCh or comorbidities, such as DE or allergic conjunctivitis. Moreover, after surgical treatment for CCh, the patient’s symptoms may worsen or persist due to a misdiagnosis, insufficient treatment, or concurrent ocular pathologies. Therefore, deciding the optimal surgical treatment for CCh refractory to medical treatment is also challenging. However, a specific method to properly evaluate these issues has not been proposed.

Some cues may help differentiate between DE and CCh. For example, CCh symptoms are exacerbated by down-gazing or vigorous blinking, while aqueous-deficient DE (ADDE) worsens on up-gazing when the interpalpebral exposure zone increases^1,17,18^. Moreover, CCh should be considered in cases of recurrent subconjunctival hemorrhage or epiphora^1,19,20^. Nevertheless, determining the primary cause of the symptoms is difficult, especially without a standardized method. This study identified a positive correlation between tear MMP-9 positivity identified using a common POC test and CCh severity, which is a considerably meaningful step towards a reliable CCh assessment test.

We identified a positive correlation between CCh severity and the semi-quantitative band density obtained in the MMP-9 POC test. Tear MMP-9 originates from the corneal epithelium, fibroblasts, and lacrimal glands^21–24^. Therefore, a highly positive MMP-9 result can be interpreted as suspicious regarding CCh-induced ocular surface inflammation if other ocular surface diseases have not been identified. Future studies could build on our findings by employing a longitudinal design to monitor treatment efficacy using MMP-9 as a biomarker. For example, a prospective study could measure tear MMP-9 levels before and after various therapeutic interventions—whether medical or surgical, as previously observed through quantitative enzyme-linked immunosorbent assay (ELISA) testing^10^. Tracking changes in MMP-9 positivity and semiquantitative grading over time would not only help confirm that treatment reduces ocular surface inflammation but also offer an objective parameter to gauge clinical response. Moreover, future research could explore whether serial MMP-9 measurements might guide clinical decision-making. For instance, persistently elevated MMP-9 levels despite initial medical therapy could prompt earlier consideration of surgical interventions in patients with CCh. By correlating changes in MMP-9 levels with patient-reported symptoms and clinical signs, such studies could refine treatment algorithms and ultimately lead to more personalized and effective management strategies.

Patients with ADDE commonly have corneal epithelial defects that can be the sole reason for elevated MMP-9 levels^14,25^. Therefore, we did not include patients with ADDE in this study to avoid misconstruing high MMP-9 concentrations and avoid false-negative results due to the extremely low tear volume in this patient population. In addition, our previous study found that the colorimetric reaction of the MMP-9 POC test was significantly reduced and delayed when the tear volume decreased^13,26^. On the other hand, since EDE can disrupt tear biomarkers, including MMP-9, we initially expected a significant difference in MMP-9 positivity between the EDE and NC groups; however, no statistical significance was observed. Nonetheless, our findings do not imply that EDE and NC are equivalent in distinguishing CCh(-) from CCh(+). It is well established that CCh is strongly associated with EDE, as previous studies^27,28^ have demonstrated a significant correlation between conjunctival folds and tear film instability, often attributed to a compromised lipid layer, which may, in turn, lead to dysregulation of tear biomarkers such as MMP-9. Given that MMP-9 is an inflammatory rather than a CCh-specific biomarker^22^, this correlation between CCh and EDE results in the MMP-9 test having low sensitivity and specificity as a diagnostic tool, as reflected in our findings.

This study has some limitations. First, we did not incorporate meibomian gland parameters into our inclusion or exclusion criteria, which means that meibomian gland dysfunction could have influenced the results in both the EDE and CCh groups in our study. Second, we did not measure the actual MMP-9 concentration in the tears of the participants, nor did we perform repeated MMP-9 POC tests after medical treatments or surgery. We primarily focused on the correlation between CCh severity and the semi-quantitative MMP-9 POC test results. Thus, our results should be carefully interpreted. Third, only a single test was conducted for the measurement of TBUT, just before measuring the ocular staining score, to minimize the influence on subsequent examinations, as repeated TBUT measurements could affect the tear concentration of MMP-9. Fourth, although our normal group had OSDI scores greater than 13, which could suggest the presence of DED, we did not classify them as having DED. This decision was consistent with prior research^29,30^, which indicates that OSDI alone is not sufficient to diagnose clinically evident DED. Both symptoms and objective signs must be considered, and an OSDI score ≥ 33 is typically used to define severe DED.

While the MMP-9 POC test can detect ocular surface inflammation by measuring MMP-9 levels, its moderate sensitivity and specificity, along with challenges in sample collection, cost, and the non-specific nature of the biomarker, limit its application as a routine primary diagnostic tool for CCh. In clinical practice, when a condition has an already well-established clinical diagnosis, such adjunctive tests are best reserved for cases where they may help monitor disease progression or the response to therapy rather than serve as the primary method of diagnosis.

In conclusion, we identified a positive correlation between CCh severity and the semi-quantitative MMP-9 POC test results. This result suggests that the MMP-9 POC test could be a potential diagnostic indicator of CCh severity and a suitable assessment tool for in-office follow-up CCh evaluations. Integrating MMP-9 monitoring into treatment protocols may serve both as a biomarker for evaluating the efficacy of anti-inflammatory or surgical treatments and as a decision-making tool to tailor interventions based on the individual patient’s inflammatory status.

## Methods

### Participants and ethical considerations

The Keimyung University Dongsan Medical Center Institutional Review Board (IRB no. DSMC 2021-04-019) approved this study, which followed the tenets of the Declaration of Helsinki. Informed consent was obtained from all participants after explaining the study in detail. This prospective cross-sectional study was performed from May 1, 2021 to October 4, 2021. This study was performed at Keimyung University Dongsan Medical Center and all participants were patients of the center. All study participants were randomly tested on only one of two eyes, with laterality determined using a randomization table generated in Excel.

### Participant classifications

The study investigator (J.L.) collected ocular surface parameters from one eye of the enrolled participants. The participants were then categorized based on the following criteria (Table 2): (1) the presence of conjunctival folds, (2) a Schirmer test result of ≥ 7.0 mm after 5 min, and (3) a tear break-up time (TBUT) of ≤ 7 s, based on “New Korean Guidelines for the Diagnosis and Management of Dry Eye Disease”^31^. Participants who did not meet these criteria except the Schirmer test were allocated to the normal control (NC) group. Participants with a TBUT < 7 s, a Schirmer test result of > 7.0 mm, but no conjunctival folds were categorized into the evaporative dry eye (EDE) group. Finally, participants with prominent conjunctival folds during the slit-lamp microscopy exam and a Schirmer test result of > 7.0 mm were classified into the CCh group.

**Table 2:** Classification criteria for normal eyes and eyes with evaporative dry eye or conjunctivochalasis.

Parameters	Groups		
	Normal	EDE	CCh
TBUT ≤ 7 s	–	+	Variable
Schirmer ≥ 7.0 mm per 5 min	+	+	+
Conjunctival folds	–	–	+ (4 scales)

CCh: Conjunctivochalasis, EDE: Evaporative dry eye, TBUT: Tear break-up time.

The exclusion criteria were a Schirmer’s test result of < 7.0 mm after 5 min as low tear volume could induce false-negative results or a considerably low colorimetric reaction and result band in the POC test^13^; lacrimal drainage disorders (e.g. punctal stenosis, canalicular anomalies, and nasolacrimal duct obstruction), temporary punctal occlusion within 3 months, or patent permanent punctal occlusion within 6 months; ocular surgery within the previous 6 months or ocular trauma during the last 3 months; concomitant ocular surface diseases, e.g. acute allergic conjunctivitis; systemic diseases, e.g. diabetes and autoimmune disorders, which can impact the ocular surface; use of systemic or topical anti-inflammatory medications; anti-glaucoma eyedrop use; and contact lens use within the prior 72 h.

### Ocular surface parameter and severity of conjunctivochalasis assessments

Ocular surface parameter assessments were performed in the following order to minimize interference between clinical examinations: (1) OSDI questionnaires^32,33^, (2) TMH by anterior segment optical coherence tomography (OCT), (3) Schirmer I test, (4) a tear MMP-9 POC test using InflammaDry^®^ (Quidel), (5) TBUT, and (6) OSS using the Oxford scheme.

The participants were asked to complete OSDI questionnaires, which comprised 12 questions related to visual disturbance, eye discomfort, and visual function, before ophthalmologic tests. TMH was measured following a previously described method^26,34^ using a swept-source OCT device (DRI OCT Triton; Topcon, Tokyo, Japan) by a single, experienced examiner (S.J.Y.). Vertical cross-section images of the enrolled eye were obtained after voluntary blinking in a dark room. TMH was defined as the distance between the cornea–meniscus junction and the lower eyelid–meniscus junction. The Schirmer I test was used to assess tear production, performed by placing a standard Schirmer tear^®^ filter-paper strip (EagleVision, Memphis, TN) over the lateral fornix of an unanaesthetised eye for 5 min and measuring the moisture zone from the tears.

**Fig. 4:**
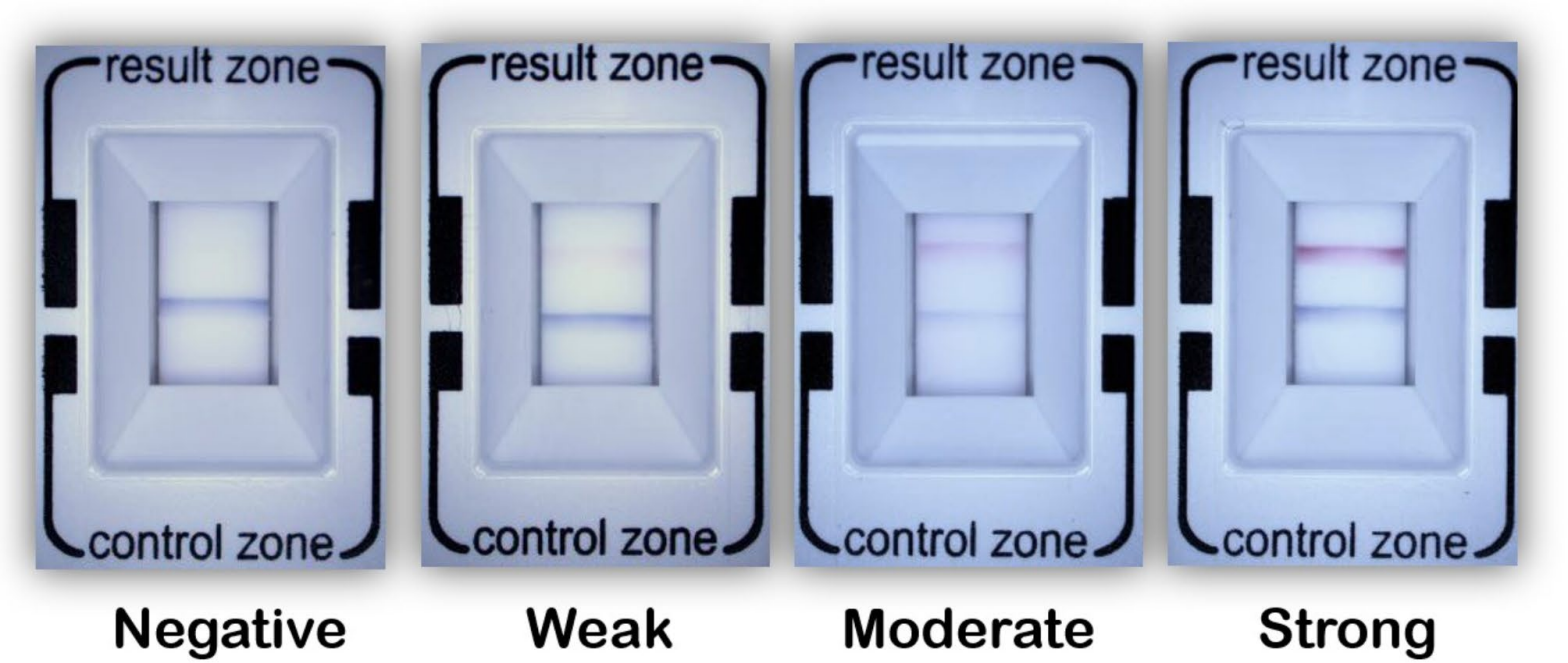
Representative images of the tear matrix metalloproteinase (MMP)-9 point-of-care test. The test results were categorized into four grades based on band density: negative, weakly positive, moderately positive, and strongly positive.

The tear MMP-9 immunoassay test was conducted following the previously established protocol^7^. The band density from the tear MMP-9 POC test was semi-quantitatively evaluated in four grades (negative, weakly positive, moderately positive, and strongly positive) (Fig. 4) or qualitatively evaluated (positive/negative) 10 min after a single examiner (J.H.J.) performed the InflammaDry assay following the manufacturer’s instructions. To ensure consistent evaluation of the test line’s band density, a photograph of the result window was taken under identical conditions using a slit-lamp biomicroscope equipped with a single-lens reflex camera (Canon EOS 700D, settings: ISO 400, shutter speed 1/200 sec; Canon USA, Melville, NY, USA). The qualitative evaluation was derived from the semi-quantitative assessment, classifying “weakly positive” results as negative. This approach was taken to account for potential bias introduced by the investigator’s detection limit and interference from medications^13^.

The TBUT and OSS were determined using a wetted fluorescein strip (Haag-Streit AG, Koenig, Switzerland) touched into the lower inferotemporal palpebral conjunctiva, observed by a yellow-filtered slit-lamp microscope by a single examiner (J.H.J.). After blinking two or three times, the interval from the last blink to the appearance of tear break-up spots on the corneal surface was recorded. Corneal and conjunctival staining were checked using fluorescein staining, and the score was measured based on the Oxford grading system. The staining scores ranged from 0 to 5 for each panel and 0–15 for the total exposed interpalpebral conjunctiva and cornea^35^. The conjunctival stain scores were evaluated on the nasal and temporal sides of the eye. CCh was assessed based on the Yokoi classification, focusing on the central (under cornea) and adjacent nasal and temporal 1/2 conjunctival areas. In brief, grade 0 indicates no fold; grade 1 represents a fold visible only upon forced blinking; grade 2 indicates a fold visible without forced blinking, lower than the TMH; and grade 3 signifies a prominent fold visible without forced blinking, higher than the TMH^36,37^.

### Data handling and statistics

Statistical analyses were performed using SPSS version 12.0 (IBM, Armonk, NY). P-values < 0.05 were statistically significant. The data were described using means ± standard deviations. Analyses related to semi-quantitative or qualitative positivity of the tear MMP-9 POC test, sex, and laterality were performed using a χ^2^ test followed by a Fisher’s exact test. Spearman’s correlation analysis was used to evaluate the correlation between tear MMP-9 positivity, CCh severity, and age. Paired-T tests were performed to compare ocular surface parameters between the CCh-negative [CCh(–)] and CCh-positive [CCh(+)] groups. Finally, a one-way analysis of variance followed by a Tukey honestly significant difference (HSD) test was used to evaluate the difference among the NC, EDE, and CCh groups.

## Data Availability

The datasets used and/or analysed during the current study available from the corresponding author on reasonable request.
